# Prize Essay of the Louisiana Medico-Chirurgical Society

**Published:** 1846-08

**Authors:** 


*Prize Essay of the Louisiana Medico-Chirurgical Society.*—We are requested to announce to the profession, that at a recent meeting of this Society, it was resolved to offer a gold medal, of the value of one hundred dollars, for the best Essay *on Strictures of the Urethra, with their treatment.* This prize is offered to the competition of the profession in all countries; but the essays must be written in the English or French language. The communications must be accompanied with a letter and corresponding mottoes to the President of the Louisiana Medico-Chirurical Society, New Orleans, La., and should be received by the first day of February, 1847.

The medical press throughout the country is respectfully requested to give publicity to this notice.

Here is the offer of a splendid prize, and we doubt not it will call forth the competition of great talent. The prize will be awarded at the anniversary meeting of the Society, on the first Wednesday of April, 1847.—*New Orléans Medical and Surginal Journal.*

